# Imaging of amyloid, tau and synaptic dysfunction in the brains of type 2 diabetic rats ‐ a multitracer PET study

**DOI:** 10.1002/alz.083556

**Published:** 2025-01-09

**Authors:** Yanyan Kong, Chuantao Zuo, Fang Xie, Jianfei Xiao, Qi Huang, Yihui Guan, Ruiqing Ni

**Affiliations:** ^1^ PET Center, Huashan Hospital, Fudan University, Shanghai, Shanghai China; ^2^ Department of Nuclear Medicine and PET Center, Huashan Hospital, Fudan University, Shanghai China; ^3^ Huashan Hospital, Fudan University, Shanghai, Shanghai China; ^4^ PET Center, Huashan Hospital, Fudan University, Shanghai China; ^5^ Huashan Hospital, Shanghai, shanghai China; ^6^ University of Zurich, Zurich, Zurich Switzerland

## Abstract

**Background:**

Type 2 diabetes mellitus (T2DM) is associated with a greater risk of Alzheimer's disease (AD). Synaptic impairment and protein aggregates have been reported in the brains of T2DM rodent models. Here, we assessed the changes in synaptic vesicle 2A (SV2A), amyloid‐β, and tau that are featured pathologies in AD in T2DM rats in vivo.

**Method:**

Positron emission tomography (PET) using [^18^F]SynVesT‐1 (SV2A), [^18^F]flumazenil (GABA_A_ receptor), [^18^F]florbetapir (amyloid‐β), [^18^F]APN‐1607 (tau), was carried out in 12‐month‐old diabetic Zucker diabetic fatty (ZDF) and Sprague‒Dawley (SD) rats. Immunofluorescence staining as well as proteomic profiling and pathway analysis were performed on the brain tissues of ZDF and SD rats.

**Results:**

Reduced cortical [^18^F]SynVesT‐1 uptake were observed in 12‐month‐old ZDF rats compared to SD rats. No difference was observed in the [^18^F]florbetapir and [^18^F]APN‐1607 uptake in the brains of 12‐month‐old ZDF and SD rats. Immunofluorescence staining revealed Thioflavin S‐negative, phospho‐tau‐positive inclusions in the cortex and hypothalamus of ZDF rats, without amyloid‐beta deposits. Proteomic analysis further demonstrated downregulated synaptic‐related proteins pathways in the hippocampus of ZDF rats compared to SD rats.

**Conclusion:**

These findings provide in vivo evidence for synaptic impairment in the brains of aged T2DM ZDF rats.

